# Peste des petits ruminants in large ruminants, camels and unusual hosts

**DOI:** 10.1080/01652176.2020.1714096

**Published:** 2020-01-25

**Authors:** Aziz-Ul- Rahman, Kuldeep Dhama, Qasim Ali, Irshad Hussain, Muhammad Oneeb, Umar Chaudhary, Jonas Johansson Wensman, Muhammad Zubair Shabbir

**Affiliations:** a University of Veterinary and Animal Sciences, Lahore, Pakistan; b Division of Pathology, ICAR-Indian Veterinary Research Institute, Izzatnagar, India; c The Roslin Institute, University of Edinburgh, Edinburgh, UK; d Department of Clinical Sciences, Swedish University of Agricultural Sciences, Uppsala, Sweden

**Keywords:** Peste des petits ruminants, large ruminants, camels, unusual hosts, cross-species transmission, *Culicoides imicola*, disease eradication

## Abstract

Since its first report in 1942, peste-des-petits-ruminants virus (PPRV) has caused several epidemics in a wide range of susceptible hosts around the world. In the last 30 years, the evidence of natural and experimental infections and virus isolation were reported from novel but unusual hosts such as camel, cattle, buffalo, dogs, Asiatic lion and pigs. In addition, PPRV in a potential vector, biting midges (*Culicoides imicola*), has been reported. Either presented as clinical and/or subclinical infections, the presence of the virus in an extended range of susceptible hosts highlights the cross-species transmission and supports the hypothesis of an endemic circulation of PPRV among susceptible hosts. However, the potential role of large ruminants, camels and unusual hosts for PPRV epidemiology is still obscure. Therefore, there is a need for molecular and epidemiological investigations of the disease among usual and unusual hosts to achieve the goals of disease control and eradication programmes initiated by national and international organisations, such as the FAO and OIE. This review is the first to summarise the scattered data on PPR in large ruminants, camels and unusual hosts to obtain the global scientific communities’ attention for further research on epidemiological aspects, not only in its native hosts, but also in large ruminants, camels and other unusual hosts.

## 1. Introduction

Peste des petits ruminant (PPR) caused by peste-des-petits-ruminants virus (PPRV), is classified as an Office International des Epizooties (OIE)-listed Transboundary Animal Disease (TAD). In the developing world, this virus poses a major threat to sustainable agricultural growth by causing significant economic losses due to high morbidity and mortality in sheep and goats (Banyard et al. 2014). In the acute form of infection, morbidity and case fatality rate may remain high (up to 100%) (Pope et al. 2013). PPRV is classified as a *Small ruminant morbillivirus* and belongs to the genus *Morbillivirus* in the family *Paramyxoviridae* (Amarasinghe et al. 2019). It has a close antigenic relationship with other viruses of the same genus, including rinderpest virus (RPV), measles virus (MV) and canine distemper viruses (CDV). These morbilliviruses have the propensity to cross species barriers, which highlights their potential towards inter-species transmission and novel host adaptation (Cosby 2012). For instance, CDV has exhibited the capability to target diverse species of carnivores and non-human primates including pandas, minks and rhesus monkeys (Sun et al. 2010; Sakai et al. 2013; Beineke et al. 2015). The same has also been observed for MV, which may cause infection of high virulence in new susceptible hosts, such as rhesus monkeys (Leonard et al. 2008). In the same way, it has been postulated that RPV crossed the species barrier into humans 1000–5000 years ago (Barrett 1999). Such evidence indicates the capability of morbilliviruses to cause infection by crossing species barriers from native to novel or unusual/atypical hosts. PPRV also has the propensity to cross species barriers (inter- and intra-species transmission) and has the potential to cause infection in non-native hosts (i.e., other than small ruminants) (Lembo et al. 2013; Mahapatra et al. 2015; Schulz et al. 2018).

The potential of PPRV to target a wide range of susceptible hosts enforces the need to improve disease control strategies for eventual disease repression. After the successful eradication of rinderpest by mass vaccination, the suppression of PPR is now the primary concern for the FAO and OIE, for which they have launched a progressive control program in endemic regions. However, PPRV’s propensity to cross the species barrier raises questions about the epidemiological role of all susceptible hosts in the spreading of the disease and evolutionary dynamics subsequent to novel host adaption. In this article, we have summarised the scattered data and listed the widening range of host species in which evidence of clinical and subclinical PPRV infection has been observed. This suggests that robust disease surveillance programmes may be initiated with appropriate interventions in disease endemic regions to eventually globally eradicate the disease.

## 2. Host susceptibility of PPRV

The transboundary nature of PPR is considered one of the main limitations in expanding the production of animals, particularly in enzootic regions of the world (Balamurugan et al. 2014). Besides domestic small ruminants as native hosts (Kumar et al. 2014; Aziz-ul-Rahman et al. 2016; Shabbir et al. 2018), camels (Zakian et al. 2016), large ruminant species, including cattle and water buffalo (Govindarajan et al. 1997; Lembo et al. 2013; Sen et al. 2014), a wide range of wild animals (Aziz-ul-Rahman et al. 2018) and unusual hosts, such as pigs (Schulz et al. 2018) are also considered as susceptible to PPRV infection with variable morbidity and mortality rates. Taken together, the significance of a widening host range in relation to possible disease control measures makes it also important to understand the disease potential of PPRV in large ruminants, camels and unusual hosts, particularly in disease-endemic regions.

### 2.1. Evidence in large ruminants (cattle and water buffalo)

Several studies have reported seroconversion to PPRV in cattle worldwide, and water buffalo and yaks in Asia (see Table 1 for an overview). To the best of our knowledge, only one report of clinical PPRV infection in large ruminants exists: an outbreak in domestic water buffalo (*Bubalus bubalis*) in India (Govindarajan et al. 1997). In this case, the clinical presentation was characterised by fever, conjunctival congestion, hypersalivation and depression, resembling what is seen in sheep and goats. Morbidity was roughly 13% with a very high case fatality rate (96%) that was not age-related (Govindarajan et al. 1997). The disease was experimentally reproducible in buffalo calves, thus confirming the ability of PPRV to induce clinical disease in this species.

**Table 1.** Evidence of natural and experimental infection of PPRV in large ruminants, camels and unusual hosts.

Host	Country	Year	References
**Clinical infections of PPRV in large ruminants and camels**			
Camel	Iran	2013	Zakian et al. (2016)
Camel	Sudan	2004-08, 2004	Kwiatek et al. (2011) and Khalafalla et al. (2010)
Camel	Ethiopia	1995-96, 2000-12	Roger et al. (2000) and Saeed et al. (2015)
Camel	Kenya	2016	Omani et al. (2019)
Water Buffalo	India	1995	Govindarajan et al. (1997)
**Detection of antibodies in large ruminants and camels as a result of natural exposure to PPRV**			
Cattle	Iran	Not available	Rasooli et al. (2019)
Cattle	Tanzania	2011, 2016	Herzog et al. (2019) and Lembo et al. (2013)
Camel	Kenya	Not available	Chemweno et al. (2019)
Cattle	Ethiopia	2005-2006, 2001	Agga et al. (2019) and Abraham et al. (2005)
Cattle, Yak	China	2016-17	Li et al. (2018)
Cattle	Sudan	2008-12, 2001, 2015-16, 2016-18	Haroun et al. (2002), Intisar et al. (2017), Ali et al. (2019), Hekal et al. (2019)
Camel	Sudan	2008-12, 2008, 2001, 2008-09	Haroun et al. (2002), Intisar et al. (2010, 2017), Saeed et al. (2010)
Cattle, Water Buffalo, Yak	Pakistan	2009, 2005-06, 2007, 2014	Khan et al. (2008), , Abubakar et al. (2017, 2019)
Camel	Nigeria	2011-03, 2012, 1995, 2012-13	Daneji et al. (1997), Bello (2013), El-Yuguda et al. (2013), Woma et al. (2015)
Camel	Libya	2014	El-Dakhly (2015)
Cattle, Water Buffalo	India	2011, 2009-10	Balamurugan, Krishnamoorthy et al. (2012), Balamurugan et al. (2014)
Cattle	Nigeria	2012-13	El-Yuguda et al. (2013)
Camel	Tanzania	2010	Swai et al. (2011)
Camel	India	Not available	Rajneesh and Tanwar (2011)
Cattle	Turkey	2009, Not available	Ozkul et al. (2002) andAlbayrak and Gur (2010)
Camel	Ethiopia	2001, 1995	Roger et al. (2000) and Abraham et al. (2005)
Cattle	Kazakhstan	1997-98	Lundervold et al. (2004)
Cattle	Bangladesh	1993, 1997-98	Anowar and Nadir (2004)
Cattle	Nigeria, Ghana	1993	Anderson and McKay (1994)
Camel	Egypt	Not available	Ismail et al. (1992)
**Experimental infection of PPRV in camels**			
Camel	Morocco	2018	Fakri et al. (2019)
**Experimental infection of PPRV in large ruminants**			
Cattle	Côte D'ivoire	2018	Couacy-Hymann et al. (2007)
Cattle	India	2013	Sen et al. (2014)
**Experimental infection of PPRV in unusual hosts**			
Pig	Germany	2015-2016	Schulz et al. (2018)
Pig	Nigeria	1978	Nawathe and Taylor (1979)
**Evidence of PPRV nucleic acid in unusual hosts and vectors**			
Biting midge	Turkey	2015	KU325483; https://www.ncbi.nlm.nih.gov/nuccore/ KU325483. . . . . . . . . . KU175171; https://www.ncbi.nlm.nih.gov/nuccore/ KU175171. . . . . . . . . .
Dog	India	2015	Ratta et al. (2016)
Asiatic lion	India	2007	Balamurugan et al. (2012)

Experimental infections in cattle have shown susceptibility to PPRV in this species, without inducing any clinical signs (Sen et al. 2014; Couacy-Hymann et al. 2007), similar to what is seen in field epidemiological studies. Cattle in contact with PPRV-infected goats became sub-clinically infected (Sen et al. 2014), and all four genetic lineages were able to induce seroconversion (Couacy-Hymann et al. 2007). Although PPRV persisted for at least 397 days after infection (Sen et al. 2014), there is no evidence for virus shedding in body secretions and excretions, indicating a low risk for further transmission (Sen et al. 2014; Couacy-Hymann et al. 2007). These findings support the hypothesis of large ruminants (at least cattle) being dead-end hosts for PPRV, (i.e., these species are susceptible to PPRV infection but not able to transmit the virus to other animals) (Agga et al. 2019). Although, there is not yet any evidence of virus shedding in secretions or excretions in water buffaloes, it is reasonable to assume that this occurred in the clinical outbreak reported from India (Govindarajan et al. 1997), because virus transmission between individual animals would be needed for such a devastating outcome.

Based on this evidence, it can be assumed that large ruminants may have a potential epidemiological role in transmission of PPRV to other susceptible hosts; or in the instance of dead-end hosts, be of value for PPR sero-surveillances, as an indicator of on-going virus circulation after vaccination campaigns in small ruminants.

### 2.2. Evidence in camels

Clinical PPRV infection and seroconversion in camels are continuously being reported from endemic regions in Africa, the Middle-East and Asia (Table 1). Thus far, all clinical signs including histological and pathological findings related to digestive and respiratory systems, have been found similar to general features of PPR in small ruminants (Khalafalla et al. 2010; Zakian et al. 2016). In these studies, PPRV has mainly been detected by immunocapture (ic) ELISA and/or RT-PCR from tissue samples characterised by lesions (Khalafalla et al. 2010; Kwiatek et al. 2011; Zakian et al. 2016). Moreover, abattoir studies on slaughtered animals report detection of PPRV antigens in lung tissue in animals with lesions, indicating a history of pneumonia (Saeed et al. 2015; Intisar et al. 2017). Most likely, these findings indicate a viral shedding, for example in faeces and nasal discharges, with the possibility for further virus transmission, although this has to be verified. Indeed, ocular discharges from one camel displaying clinical signs resembling PPR were found PCR-positive in a recently published study from Kenya (Omani et al. 2019), strongly indicating that camels do shed PPRV. This study characterised an outbreak in camels, referred to as “camel sudden death syndrome”, where animals displayed fever, diarrhoea, conjunctivitis with ocular discharges, loss of body condition and general weakness, thus resembling PPR in small ruminants. Sheep and goat flocks herded side by side to camels showed typical signs of PPR, and PPRV of the same lineage (III) was also detected in one of the goats (Omani et al. 2019). Contrary to these findings, a recent study observed no clinical infection of PPR in camels after experimental exposure and claimed that camels play no epidemiological role in the spread of the disease (Fakri et al. 2019). Indeed, little evidence has been shown regarding the transmission of the virus by shedding from infected camels, and there is still a controversy around the involvement of camels in the spread of the disease. Possibly in natural conditions, an animal facing stress caused by environmental change, co-infections and other factors, could lead to an impaired general condition that favours pathogens to clinically affect atypical hosts (Chapman et al. 2005). Therefore, there is a need to further investigate camel susceptibility to PPRV, in both natural and experimental conditions.

Taken together, camels do not seem to be a dead-end host, and virus transmission between sheep, goats and camels needs to be considered when controlling PPRV. Whether PPRV is the single etiological cause of clinical signs in camels, resembling PPR in sheep and goats, has yet to be proven by further studies. Clinical and subclinical PPRV infection in camels, cattle and water buffalo may indicate novel aspects on the epidemiology and pathogenesis of PPRV, with implications for the mechanism of adaptation of the virus in a new host niche. Simultaneously, although there is an unapparent clinical infection demonstrated, virus secretion has also been reported in wild camels, cattle and buffalo (reviewed in Aziz-ul-Rahman et al. 2018).

### 2.3. Evidence in unusual hosts (pigs, dogs and Asiatic lion) and potential vectors (biting midges)

It is suggested that PPRV has the potential to switch hosts as is postulated in other morbilliviruses (De Swart et al. 2012). Until now, there is a paucity of data related to the clinical infection and transmission of PPRV in unusual hosts including pigs, dogs and Asiatic lion. An experimental investigation revealed a subclinical infection of PPRV in pigs with no shedding and transmission of viruses to a native host (Nawathe and Taylor 1979). On the other hand, recent experimental investigations highlighted the epidemiological role of pig and wild boar in inter- and intra-species transmission of PPRV (Schulz et al. 2018). As a virus amplifier, pigs pose potential health risks for other susceptible species and may create hurdles to the eradication of the disease, particularly in disease-endemic countries where free-roaming pigs and communal grazing of sheep and goats are common. Future investigations are imperative to explore the possible role of pigs in virus transmission to domestic small and large ruminants, wild ungulates and other susceptible species. Owing to persistent transhumance and pastoralism among susceptible species, the spill-over of PPRV has utmost significance in cross-species transmission, which leads to constraints in disease eradication.

In addition, two reports highlight the susceptibility of PPRV for Asiatic lion and dogs, because of the detection of PPRV genomes in routine screenings of pooled tissue samples and nasal swabs, respectively (Balamurugan, Sen et al. 2012; Ratta et al. 2016). Although PPRV has a close antigenic relationship with CDV, which is a common pathogen for dogs and lion, the PPRV-positive Asiatic lion (Balamurugan, Sen et al. 2012) and one of the three PPRV-positive dogs (Ratta et al. 2016) were found negative for CDV. Interestingly, two of the PPRV-positive dogs had gastroenteritis, while the other was suspected of suffering from canine distemper. However, the detection of PPRV antigens/nucleic acids in nasal swabs and tissues from dog and Asiatic lion is probably due to these animals having been fed PPRV-infected sheep/goat meat or contaminated fomites. In PPR-endemic countries, dogs usually live in companionship or in close proximity to domestic small ruminant farming, thereby, there is a likely chance of transmission of viruses from infected hosts to dogs.

Recovery of the PPRV gene segments from biting midges (C*ulicoides imicola*) in Turkey indicate the possibility of virus transmission through vectors (NCBI accession numbers KU325483 & KU175171), although further studies are needed to confirm these results and to investigate whether biting midges are competent or mechanical vectors.

Although, both cellular receptors (Nectin-4 and SLAM) have significance for host adaptation of PPRV, the conserved PPRV sequences obtained from domestic small ruminants and unusual host origin indicate that this virus has the potential to switch host without necessary mutations (Rahman et al. 2019). Such evidence suggests the possibility of crossing the species barrier. However, further serological-and molecular-based evidence is necessary. Both large ruminants and unusual hosts might be assessed to detect an eventual change in viral host–pathogen interaction for a wide range of species. The detection of PPRV nucleic acids in dogs, Asiatic lion and biting midges cannot be overlooked and may be of epizootiological significance in studying the host diversification capacity of viruses, which may also require new intervention strategies to control PPRV infection.

## 3. Cross-species spill-over/“jumping” of virus

Natural or experimental PPRV infection has now been shown to induce clinical disease affecting camels (Khalafalla et al. 2010; Kwiatek et al. 2011; Zakian et al. 2016) and unusual hosts such as pigs (Nawathe and Taylor 1979; Schulz et al. 2018). Subsequent to the occurrence of likely spill-over events, such evidence indicates the potential of the virus to affect a wide range of hosts, particularly in disease-endemic regions. Such spill-over events are most likely to occur as a result of interactions between PPRV-infected small ruminants and other susceptible hosts during epidemic or endemic conditions and may act as a foundation for further inter-species transmission, particularly in livestock-dense endemic regions. PPRV has previously been reported in other species, such as populations of several species of wild mountain goat (genus *Capra*), and is most likely due to spill-over from domestic small ruminants (Abubakar et al. 2011). Thus, the viral-host jumps are not uncommon for PPRV and associated with provoking hurdles in disease eradication worldwide. Similar findings of viral transmissions have previously been reported from infected sheep and goats to wild ungulates in Saudi Arabia (Frölich et al. 2005). Recently, PPRV has been reported from the four-horned antelope (*Tetracerus quadricornis*), an endangered bovid species of India. During the outbreak, 20 animals died and this was attributed to PPRV lineage IV (Jaisree et al. 2018). Further investigations are needed to ascertain the status of PPRV among endangered animals like the four-horned antelope or Chowsingha. In these scenarios, direct or indirect contact with infected domestic small ruminants may transmit the virus to other hosts. Together this evidence further substantiates the assumption that, while living in close proximity to each other, particularly in a developing country setting, there are several chances for cross-species transmission, either from small ruminants to large ruminants or to unusual hosts (Lembo et al. 2013; Mahapatra et al. 2015; Schulz et al. 2018). With reference to the genetic makeup, the viral strains responsible for infection, in both small and large ruminants, exhibit a marked genetic relatedness (Rahman et al. 2019). Despite these facts, the potential transmission of PPRV, and factors involved in disease epidemiology among domestic small and large ruminants and unusual host populations in disease-endemic regions, are largely unknown.

## 4. Prospective in disease eradication

PPR is an eminent disease and eradication needs to be prioritised for poverty alleviation and food security, because of the high morbidity and mortality in small ruminants commonly owned by resource-constrained farmers. However, to further support disease control policies, there is need for further research on several epidemiological features, such as transmission dynamics among known and/or novel hosts raised either under similar or different production systems (Jost et al. 2007). In fact, mass die-offs in small and large ruminants in PPR endemic regions of developing countries are severely affecting the livestock productivity and poor communities’ livelihoods. Hence, an analytical study about the incidence of the disease in various susceptible hosts would be extremely useful and elicit widespread interest by providing sufficient additional information, especially in the epidemiology of the disease (Dhar et al. 2002). Moreover, future studies on the genetic characterisation of current prevailing PPRV strains and their epizootiology, related to susceptible hosts, would be helpful in the implementation of disease control policies and also to determine if large ruminants, camels and unusual hosts play any role in the endemicity of PPR.

The evidence of PPR in large ruminants, camels and other unusual hosts raises concerns for the strengthening of disease surveillance and control strategies. This is important in areas where the disease is enzootic and animal density, including for small and large ruminants, is high, such as in Africa and Asia. Since spatial and temporal heterogeneities exist in animal population density, and subsequent differences in susceptibility to PPR infection, disease eradication seems to be a long process that cannot solely rely on mass vaccination. Considering the susceptibility and role of unusual/atypical hosts in the spread of disease, there may be various factors influencing the hindrance of the global eradication of PPR, particularly in disease-endemic countries. Susceptibility of unusual hosts to PPR and their potential to spread disease, has been shown (Schulz et al. 2018). Therefore, the lack of disease surveillance, monitoring and proper diagnosis in these hosts may be causing the delay of disease eradication. Since data related to disease epidemiology and the contribution of large and unusual animals in disease transmission is scarce, efforts should be made to further research on spatial and temporal disease transmission in endemic countries. Effective and thorough targeting of a wide range of hosts (host heterogeneities) was one of the key factors that enabled the eradication of rinderpest. Furthermore, spatial and temporal heterogeneities in susceptible hosts, and their sensitivity to PPRV and role in disease epidemiology, make the situation even more complicated for disease eradication. As such, epidemiological modelling may be useful in these circumstances, to help in deciding on the best strategy for combating this devastating disease. Therefore, a good knowledge of species susceptibility and other epidemiological parameters is essential for the success of a PPR eradication programme. Additionally, realistic models taking host heterogeneities into account still require further research efforts in disease epidemiology and modelling.

In most endemic settings, small and large ruminants and camels are raised together, which easily leads to the occurrence of epidemics. Thereby, there is a dire need to devise novel disease control initiatives/strategies and standardised protocols involving disease surveillance and outbreak management in all susceptible hosts living together in a single unit production setting (combined small and large animal farming). A stronger knowledge of animal dynamics, single animal unit production, and management practices and animal movements (trade, transhumance), will be a critical condition for success. Serological surveys provide crucial information on the possible presence of any disease in unusual hosts, because information on active disease presence may not be available due to poor accessibility to these animals. A new framework, including one that investigates atypical hosts in disease surveillance, is needed by the concerned national and international authorities for a stage-wise progressive control of PPR. Such expansion of disease screening, on the basis of sample availability from all susceptible hosts including atypical hosts, will surely strengthen eradication modelling in the final stages of an eradication programme. A lack of proper awareness on the role of susceptibility of atypical hosts to PPRV in veterinary and para-veterinary personnel, may create a major obstacle towards the progressive control of PPR in disease endemic regions.

Indeed, continuous disease surveillance, along with realistic epidemiological modelling (which needs the engagement of local communities; Fischer et al. 2016), could facilitate choosing the best context-specific strategy, including vaccination frequency, spatial setting of vaccinations, target species, and quick and timely diagnosis. In this regard, either based upon immunological response or direct antigen detection, several specific and sensitive laboratory methods with rapid turn-around are available for confirmation of PPRV infection (Santhamani et al. 2016; Mahapatra et al. 2019). In fact, accurate and advanced disease diagnostic techniques and vaccine constructs targeting the prevalent strain are essential for effective disease control.

To explore the impact of PPR on nature conservation, in terms of the involvement of a wide range of species, a regular serological and clinical surveillance of PPR in all susceptible hosts should be employed. Regarding this subject, the FAO and OIE are currently working on strategies to make progress in international control under the PPR Global Eradication Programme (GEP-PPR), over the coming years and have set the goal of eradicating the disease by 2030. This review article will undeniably assist all scientific communities working on disease eradication and towards sustainable improvement in economy by livestock production. Additionally, these results will strengthen the livelihood resilience of rural societies and benefit food and nutrition security. Taken together, future regional and global programmes for the control of PPR will need to consider and resolve the issues raised here, and wildlife and livestock managers or authorities in PPR endemic countries may need to encourage the protective vaccination of small and large ruminants surrounding wild populations.

Disease surveillance programs need to be further extended and strengthened to include usual and unusual hosts. Awareness of disease in large ruminants and unusual hosts should be imparted to farmers, particularly those living in disease-enzootic regions. In this regard, particular attention should be given by national and international authorities (such as the FAO/OIE) under the programme on Global Framework for the Progressive Control of Transboundary Animal Diseases (GF-TADs). This will enable better comprehension coordination among local veterinary authorities and the subsequent implementation of disease control/eradication programmes.

## Conclusion

Besides usual hosts (small ruminants, wild ungulates), PPRV has the potential to infect large ruminants, camels and other unusual hosts. Cross-species transmission, from small to large ruminants/unusual hosts may be prevented by extensive and further-strengthened disease surveillance, coupled with appropriate diagnostics and vaccine constructs. Therefore, for effective disease eradication programmes worldwide (GEP-PPR) under the GF-TADs programme, it is imperative to explore the role of large ruminants and unusual hosts to better elucidate disease epidemiology, particularly in disease-enzootic regions.
